# Numerous genetic loci identified for drought tolerance in the maize nested association mapping populations

**DOI:** 10.1186/s12864-016-3170-8

**Published:** 2016-11-08

**Authors:** Chunhui Li, Baocheng Sun, Yongxiang Li, Cheng Liu, Xun Wu, Dengfeng Zhang, Yunsu Shi, Yanchun Song, Edward S. Buckler, Zhiwu Zhang, Tianyu Wang, Yu Li

**Affiliations:** 1 Institute of Crop Science, Chinese Academy of Agricultural Sciences, Beijing, 100081 China; 2 Institute of Food Crops, Xinjiang Academy of Agricultural Sciences, Urumqi, 830000 China; 3 Institute for Genomic Diversity, Cornell University, Ithaca, NY USA; 4 USA Department of Agriculture-Agricultural Research Service, Ithaca, NY USA; 5 Department of Crop and Soil Sciences, Washington State University, Pullman, WA USA

**Keywords:** Maize, Nested association mapping population, Drought tolerance, Joint linkage mapping, GWAS, Candidate gene

## Abstract

**Background:**

Maize requires more water than most other crops; therefore, the water use efficiency of this crop must be improved for maize production under undesirable land and changing environmental conditions.

**Results:**

To elucidate the genetic control of drought in maize, we evaluated approximately 5000 inbred lines from 30 linkage-association joint mapping populations under two contrasting water regimes for seven drought-related traits, including yield and anthesis-silking interval (ASI). The joint linkage analysis was conducted to identify 220 quantitative trait loci (QTLs) under well-watered conditions and 169 QTLs under water-stressed conditions. The genome-wide association analysis identified 365 single nucleotide polymorphisms (SNPs) associated with drought-related traits, and these SNPs were located in 354 candidate genes. Fifty-two of these genes showed significant differential expression in the inbred line B73 under the well-watered and water-stressed conditions. In addition, genomic predictions suggested that the moderate-density SNPs obtained through genotyping-by-sequencing were able to make accurate predictions in the nested association mapping population for drought-related traits with moderate-to-high heritability under the water-stressed conditions.

**Conclusions:**

The results of the present study provide important information that can be used to understand the genetic basis of drought stress responses and facilitate the use of beneficial alleles for the improvement of drought tolerance in maize.

**Electronic supplementary material:**

The online version of this article (doi:10.1186/s12864-016-3170-8) contains supplementary material, which is available to authorized users.

## Background

Maize (*Zea mays* ssp. *mays* L.) is one of the three most important cereal crops and has the second highest cultivation area worldwide (http://faostat.fao.org). However, the productivity of this crop is frequently reduced in response to drought stress. Traditional breeding has shown limited progress in improving maize drought tolerance under water-limited conditions; therefore, determining how maize responds to drought stress will provide new tools for the genetic improvement of crop yields in arid environments [1].

Grain yield (GY) under water stress is a primary trait used to evaluate the degree of drought tolerance in maize [2]. Certain secondary traits associated with drought tolerance, such as the anthesis-silking interval (ASI), plant height (PH) and grain yield components, are highly correlated with drought tolerance and exhibit increased heritability [3–7]. Hence, these traits have been used to improve the selection efficacy for drought tolerance in plant breeding and identify the underlying functional quantitative trait loci (QTLs)/genes that control drought tolerance [8].

Previous studies have reported the use of linkage analyses and/or association mapping to identify drought-related quantitative trait loci. QTL mapping for GY and agronomic traits associated with drought tolerance have been conducted in a number of different bi-parental populations under well-watered (WW) and water-stressed (WS) conditions [4, 9–12]. The QTLs for drought tolerance identified in maize are available at http://www.maizegdb.org and http://www.plantstress.com, and several researchers have collected published QTL results, and data associated with QTLs for drought stress or drought tolerance obtained in different populations were used to conduct QTL meta-analyses [11, 13–15] to identify consensus QTLs and shrink the QTL confidence interval. However, the QTL studies with bi-parental populations can only detect two alleles and have limited mapping power and resolution.

Furthermore, association mapping based on linkage disequilibrium has been used to identify the causal genes affecting GY and agronomic traits associated with drought responses. Lu et al. [16] identified several single nucleotide polymorphisms (SNPs) associated with ASI and PH under drought tolerance in 305 diverse inbred lines genotyped using a 1536 SNP array. Xue et al. [17] identified 42 drought-associated SNPs for nine agronomic traits using a 350 tropical and subtropical maize association panel and data on 56 K SNPs. Thirunavukkarasu et al. [18] used 240 accessions of subtropical maize and 56,110 SNPs to conduct association analyses for seven agronomic traits, including ASI, grain yield and five-grain yield component traits under WW and WS conditions, and their results indicated that 61 SNPs were significantly associated with drought tolerance. These drought studies were performed using small association panels and low marker densities that were unable to identify the global beneficial alleles for drought tolerance.

Currently, two publically available maize genetic resources called nested association mapping (NAM) populations have been developed in the US (US-NAM) and China (CN-NAM). The US-NAM population consists of 25 bi-parental families, including approximately 5000 recombinant inbred lines (RILs) [19]. The CN-NAM population consisted of 11 bi-parental families, including approximately 2000 RILs [20]. These populations provide increased mapping resources to successfully dissect the genetic architecture of different complex agronomic traits, such as the flowering time [21], leaf architecture [22], male and female inflorescences [23], kernel components traits [24], plant height [25], stalk strength [26], etc. Using these NAM populations, we systematically dissected the genetic loci controlling maize drought tolerance using a joint linkage analysis and genome-wide association studies (GWAS). In addition, both NAM populations were sequenced using the Genotyping-By-Sequencing (GBS) method, and high-density recombination maps were constructed based on the GBS-obtained data [20]. These methods improved the mapping resolution.

In the present study, two sets of NAM populations were used to determine the phenotype of seven drought-related traits under well-watered (WW) and water-stressed (WS) conditions. Joint linkage QTL mapping was performed to detect the genomic regions that control maize drought tolerance under different water regimes. GWAS was conducted to identify the candidate genes that were significantly associated with seven drought-related traits. Furthermore, the candidate genes were validated using the RNA-seq data of the inbred line B73 obtained under the WW and WS conditions. In addition, cross-validated genomic predictions were performed to assess the accuracy of predicting drought-related traits under the two water regimes.

## Results

### Analysis of phenotypes under the WW and WS conditions

The heritability of the seven drought-related traits and average phenotypic performance based on the BLUP values is shown in Additional file 1: Table S1 for CN-NAM and Additional file 2: Table S2 for US-NAM. Significant differences were observed for the means of all of the traits within the two NAM populations under the WW and WS environments using the *F* test. The results suggested that drought stress at the flowering stage had different effects on drought-related traits. The average grain yield per plant (GYPP) in the CN-NAM population decreased by 28 % under WS, whereas it decreased by 66 % decrease in the US-NAM population under WS. As expected, the average ASI in the two NAM populations was longer under WS than under WW because the ASI reflects the susceptibility of different genotypes to drought stress. The estimated heritability for the seven traits of the CN-NAM population under WS and WW ranged from 49.3 to 83.5 % and from 61.7 to 89.4 %,respectively. The heritability estimates of the seven traits were all higher under WW than under WS.

The phenotypic correlations among the seven traits are listed in Additional file 3: Table S3 and Additional file 4: Table S4 for the CN-NAM and US-NAM population, respectively. Except for ASI and EL under the two water regimes, significant phenotypic correlations were observed among all the traits, and significant negative correlations were observed between the ASI and the remaining traits under both water regimes.

### Joint linkage mapping of drought-related traits

We identified the QTLs that control drought tolerance-related traits under the WW and WS conditions using a joint linkage analysis of the CN-NAM population. We identified 8–23 QTLs for the seven drought-related traits under WW, and they explained 23.7–66.3 % of the total phenotypic variation, whereas we identified 8–20 QTLs under WS, and they explained 20.2–55.4 % of the total phenotypic variation (Table 1). A single joint QTL could explain 0.9–8.3 % of the phenotypic variation under WW depending on the trait, whereas it could explain 1.2–10.4 % of the phenotypic variation under WS (Additional file 5). Except for GYPP, other traits were detected as consensus QTLs under different water regimes, with a total of 35 consensus QTLs identified. The consensus QTLs for each trait could explain more than 50 % of the total phenotypic variation by all QTLs detected in the corresponding trait except the KNPR. The joint linkage analysis enabled the estimation of an independent allele effect for each QTL in all 11 CN-NAM families. The total QTL allele effects ranged from 88 for GYPP to 253 for HKW under WW (31–73 % alleles were significant at *P* < 0.05), whereas the number ranged from 88 for GYPP to 220 for PH under WS (27–43 % alleles were significant at *P* < 0.05).

**Table 1:** Joint linkage analysis of seven drought-related traits under the WW and WS conditions in the CN-NAM population

Trait	WW			WS			WW-WS	
	QTL number	PVE	H^2^	QTL number	PVE	H^2^	Shared QTL number	PVE_WW_/PVE_WS_
ASI	15	48.1	61.7	11	42.8	57.3	4	26.2/22.9
PH	21	57.9	87.2	20	54.7	75.9	11	45.5/41.9
GYPP	8	23.7	68.9	8	20.2	49.3	0	0/0
EL	15	53.7	81.5	11	36.1	67.2	4	34.1/24.9
HKW	23	66.3	89.4	18	55.4	83.5	11	50.9/42.0
KNPR	13	34.7	77.7	10	28.1	61.3	1	7.7/6.1
EW	13	47.3	79.5	13	34.6	56.5	4	26.5/17.6
Total	108	--	--	91	--	--	35	--

*PVE* phenotypic variation explained by all QTLs (%), *H* ^*2*^ broad-sense heritability (%), *WW-WS* common QTLs detected under WW and WS, *PVE*_*WW*_ phenotypic variation explained by common QTLs under WW (%), *PVE*_*WS*_ phenotypic variation explained by common QTLs under WS (%)

To validate the QTLs identified in the CN-NAM population, we also conducted a joint linkage analysis in the US-NAM population (Additional file 6). A total of 112 and 78 joint QTLs were detected for all of the traits in the US-NAM population under the WW and WS conditions, respectively. Among the 112 and 78 QTLs, 32 and 18 QTLs were identified under the WW and WS conditions in the CN-NAM population, respectively, and each trait under the different water conditions had consensus QTLs between the CN-NAM and US-NAM populations except for KNPR under WS (Additional file 7: Table S5).

### GWAS of drought-related traits

**Fig. 1:**
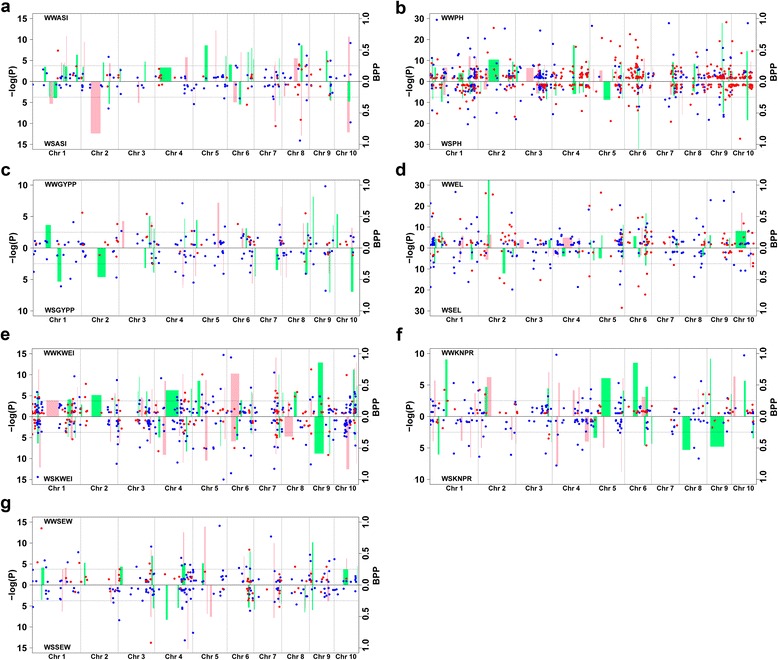
Comparison between the results of the joint linkage analysis and GWAS for the seven drought-related traits under the WW and WS conditions for the CN-NAM and US-NAM populations. The *pink* and *green* bars represent the joint QTLs for CN-NAM and US-NAM, respectively. The bar width represents the support interval of the QTLs. *Blue* and *red* dots represent the significantly associated SNPs for CN-NAM and US-NAM, respectively. **a** ASI, **b** plant height (PH), **c** grain yield per plant (GYPP), **d** ear length (EL), **e** hundred kernels weight (HKW), **f** kernel number per row (KNPR), and **g** ear weight (EW)

The genome-wide association study (GWAS) identified 1075 SNPs under WW and 795 SNPs under WS with a bootstrap posterior probability (BPP) ≥0.05 for all of the traits in the CN-NAM population (Additional file 8), and it identified 700 SNPs under WW and 448 SNPs under WS in the US-NAM population (Additional file 9). In certain cases, clusters of associated SNPs were detected within less than 100 kb of each other. The SNPs within a cluster were all identified based on a strong linkage with the same causative genes. Therefore, 100 kb was selected as a non-overlapping sliding window, and the SNPs with the highest statistical significance were selected as representatives of this window. A total of 778 SNPs under WW and 649 SNPs under WS for the CN-NAM population and 586 SNPs under WW and 359 SNPs under WS for the US-NAM population were identified in this manner (Fig. 1).

To identify the most robust associated SNPs, we further selected a BPP ≥ 0.25 as a significance threshold, which detected a total of 255 and 146 strongly associated SNPs for CN-NAM and US-NAM, respectively (Fig. 1). The GWAS results for both NAM populations were compared with both NAM joint QTL intervals. In the WW treatment, 27 % of the strongly associated SNPs identified in both NAM populations overlapped with both NAM joint QTL intervals, whereas in the WS treatment, 29 % strongly associated SNPs overlapped. A total of 221 and 179 strongly associated SNPs were detected in both NAM populations under the WW and WS conditions, respectively. Among the SNPs detected under the different water regimes, 18 associated SNPs were detected in both water regimes, which suggests that these SNPs were constitutive loci in different water environments. Ultimately, 365 strongly associated SNPs were used to identify the candidate genes associated with drought tolerance.

### Identification of candidate genes

The predicted genes close to each of the 365 underlying genes might be identified using a publicly available maize genome database (http://www.maizesequence.org), and 354 candidate genes were identified. Among the 365 SNPs, 185 SNPs were located within the coding region of the candidate genes, whereas the remaining 180 SNPs were located closer to the candidate genes, with a physical distance between the SNPs and the candidate genes ranging from 1 to 172,496 bp based on the B73 reference genome v2 (Additional file 10).

To validate the candidate genes associated with drought tolerance, we used the significantly differentially expressed genes obtained through the RNA-seq of the ovaries during flowering from the inbred line B73 under the WW and WS environments. Among the 354 candidate genes, 52 genes showed significantly different expression under the two water treatments, including 25 up-regulated genes and 27 down-regulated genes (Table 2).

**Table 2:** List of SNPs significantly associated with seven drought-related traits and the closest candidate genes with significantly differential expression between the samples under the WW and WS conditions in B73

Chr	Position (bp)^a^	BPP	Inside QTL^b^	Trait affected	Gene ID	Proximity of SNP to gene (bp)	Gene function	DEG^c^
1	4606329	0.28	-	HKW	GRMZM2G341934	1721	Peroxidase superfamily protein	0.05
1	27627382	0.27	+	PH	GRMZM2G111324	Inside gene	O-Glycosyl hydrolases family 17 protein	0.07
1	35465166	0.39	+	HKW	GRMZM2G164562	Inside gene	Chorismate synthase	0.56
1	154107233	0.53	-	KNPR	GRMZM2G056039	25433	Heat shock protein 70	2.01
1	163956308	0.49	-	ASI	GRMZM2G443525	130508	ADR1-like 1	3.92
1	219379659	0.25	+	HKW	GRMZM2G146278	1444	Cytochrome B561-1	0.24
1	269328425	0.36	-	EL	GRMZM2G157727	Inside gene	Phytochrome A	3.15
1	275974364	0.30	-	PH	GRMZM2G173852	590	Acyl-CoA N-acyltransferase with RING/FYVE/PHD-type zinc finger protein	1.52
1	287727328	0.57	-	PH	GRMZM2G133023	4891	Stem-specific protein TSJT1	9.94
2	10624855	0.26	-	EL	GRMZM2G098239	Inside gene	HXXXD-type acyl-transferase family protein	0.05
2	19262986	0.42	-	KNPR	GRMZM2G162333	1756	Pectin lyase-like superfamily protein	0.02
2	236797598	0.38	-	GYPP	GRMZM2G016677	229	Photosystem II subunit P-1/PsbP	0.41
3	17258723	0.56	-	EW	GRMZM5G871336	Inside gene	Expressed protein	4.87
3	165705047	0.51	-	EL	GRMZM2G057823	17923	Aldolase superfamily protein/fructose-bisphospate aldolase isozyme	0.19
3	197321346	0.38	-	HKW	GRMZM2G021704	Inside gene	Pyrimidin 4	0.33
4	26425071	0.27	-	PH	GRMZM2G089995	Inside gene	Ethylene response factor 7/AP2 domain containing protein	4.23
4	172055239	0.62	+	EL	GRMZM2G062084	260	P-loop containing nucleoside triphosphate hydrolases superfamily protein/kinesin motor domain containing protein	0.13
4	204344848	0.38	-	HKW	GRMZM2G117064	1105	Long chain acyl-CoA synthetase 9	0.44
4	218157821	0.31	-	HKW	GRMZM2G422464	2207	HhH-GPD base excision DNA repair family protein	2.24
5	10273708	0.55	-	GYPP	GRMZM5G833140	Inside gene	CHASE domain containing histidine kinase protein	2.59
5	11908448	0.28	-	KNPR	GRMZM2G157147	Inside gene	Phosphatidylinositol-4-phosphate 5-kinase 1	0.16
5	16321609	0.36	-	ASI	GRMZM2G134980	Inside gene	Chaperone protein dnaJ	0.69
5	22556254	0.88	+	PH	GRMZM5G869246	Inside gene	Kinesin motor family protein	0.32
5	85252655	0.50	+	HKW	GRMZM2G104632	14	Glyceraldehyde-3-phosphate dehydrogenase of plastid 1	0.30
5	140093287	0.32	-	HKW	GRMZM2G060253	Inside gene	HMG (high mobility group) box protein	0.03
5	143975579	0.51	-	HKW	GRMZM2G158313	1348	Basic-leucine zipper (bZIP) transcription factor family protein	0.01
5	208637679	0.95	+	EL	GRMZM2G081214	Inside gene	Phosphate-responsive 1 family protein	6.03
6	9710805	0.26	-	KNPR	GRMZM2G055238	60720	Ureide permease 5	5.40
6	58454978	0.50	-	HKW	GRMZM2G473788	4959	Expressed protein	1.87
6	82185973	0.66	+	PH	GRMZM2G430680	Inside gene	Glucan synthase-like 12	0.34
6	85797877	0.25	+	PH	GRMZM2G365961	52	Prephenate dehydrogenase family protein	0.15
6	88935877	0.40	+	PH	GRMZM2G150302	13001	Nucleotide-diphospho-sugar transferases superfamily protein/glycosyltransferase family 43 protein	0.41
6	119695193	0.50	-	HKW	GRMZM2G368678	Inside gene	Binding/expressed protein	1.80
6	121945548	0.49	-	EL	GRMZM2G142409	Inside gene	VIRB2-interacting protein 2/reticulon domain containing protein	5.21
6	150839908	0.41	-	EL	GRMZM2G178797	1986	Guanylyl cyclase 1	2.11
6	155438561	0.28	+	GYPP	GRMZM2G117344	12391	Expressed protein	0.18
7	135573138	0.64	-	PH	GRMZM2G130959	140	Bug22p-like protein	2.47
7	152001464	0.27	-	EL	GRMZM2G105750	36998	ATP binding;protein kinases;protein serine/threonine kinases	3.08
7	157758647	0.33	+	HKW	GRMZM2G129354	Inside gene	ARF-GAP domain 5/GTPase-activating protein	3.00
7	160601468	0.37	+	HKW	GRMZM2G058197	Inside gene	C2H2-like zinc finger protein	1.85
8	129080393	0.61	+	ASI	GRMZM2G171781	4780	MYB domain protein 61	0.03
8	148509016	0.56	-	KNPR	GRMZM2G013625	Inside gene	Associated molecule with the SH3 domain of STAM 2	3.20
8	166653395	0.26	+	ASI	GRMZM2G143640	Inside gene	MYB family transcription factor	20.26
8	166781122	0.30	+	ASI	GRMZM5G805609	Inside gene	Glycosyl hydrolase superfamily protein	4.33
9	24062413	0.35	+	KNPR	GRMZM2G082855	565	ERECTA-like 2/receptor-like protein kinase 5 precursor	0.34
9	24062931	0.61	-	PH	GRMZM2G082855	47	ERECTA-like 2/receptor-like protein kinase 5 precursor	0.34
9	31283082	0.26	-	GYPP	GRMZM2G308595	76352	Nudix hydrolase homolog 21	8.39
9	78421440	0.32	+	HKW	GRMZM2G094497	Inside gene	ATPase, V1 complex, subunit B protein	0.58
9	133586941	0.31	-	HKW	GRMZM2G317262	252	F-box family protein	6.68
9	134721582	0.94	-	PH	GRMZM2G033846	250	Ca2 + -binding protein 1/EF hand family protein	0.05
10	6398185	0.37	+	PH	GRMZM2G057753	Inside gene	Ovate family protein 13/DUF623 domain containing protein	0.10
10	15549436	0.64	-	PH	GRMZM2G088689	Inside gene	Thiamin diphosphate-binding fold (THDP-binding) superfamily protein/dehydrogenase E1 component domain containing protein	5.47

^a^physical position based on B73 maize reference genome v2

^b^ “+” repesents that significantly assiciated SNP is located in QTL support interval; “-” repesents that significantly assiciated SNP is not located in QTL support interval

^c^differential expressed genes (DEG),expression level ratios of candidate genes between WS and WW from B73

### Genomic predictions of drought-related traits

**Fig. 2:**
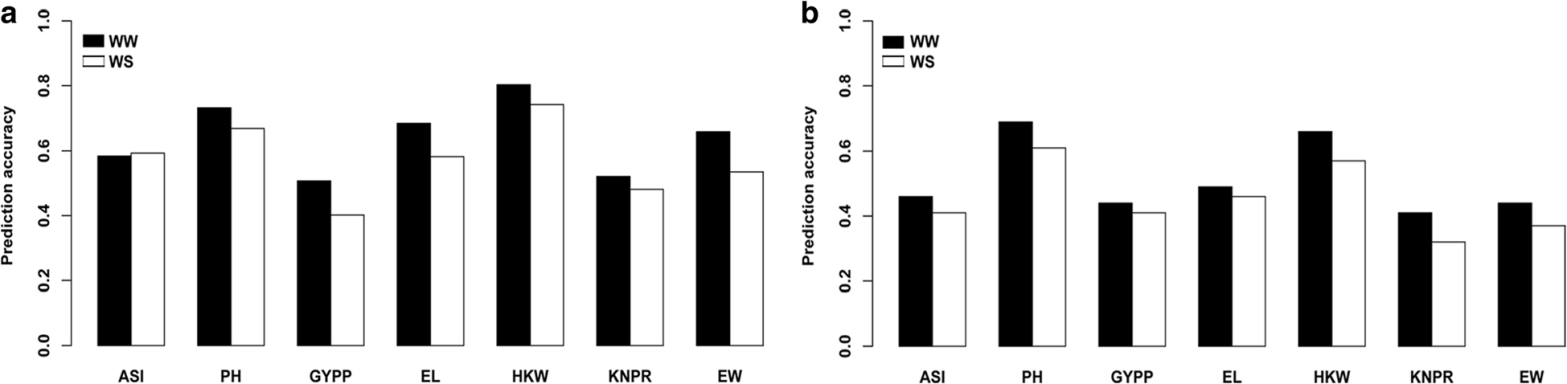
Prediction accuracy of seven drought-related traits under WW and WS conditions using genome-wide SNPs in the CN-NAM (**a**) and US-NAM (**b**) populations

Figure 2a shows the accuracy of the genomic predictions for all of the target traits evaluated under the WW and WS conditions using GBS data in the CN-NAM population. The prediction accuracy differed among all of the predicted traits for both water regimes. The accuracy values under WW ranged from 0.51 to 0.80, with a mean of 0.64, whereas the values under WS ranged from 0.40 to 0.74, with a mean of 0.57. Except for the ASI, the accuracy values of other traits under WS were consistently lower than those under WW. Reflecting differences in the heritability and genetic architecture of the target traits, the GYPP and KNPR had small prediction accuracy under the same water regime compared with other agronomic traits. In addition, we conducted genomic prediction for all traits under the WW and WS conditions in the US-NAM population (Fig. 2b). Moreover, similar trends were observed between the two populations, although lower accuracy was observed for the US-NAM population compared with the CN-NAM population.

## Discussion

In the present study, we assessed the phenotypes of seven important agronomic traits associated with drought stress for two sets of NAM populations under well-watered and water-stressed environments. Although the CN-NAM population, which included 1972 lines, was evaluated for only 2 years at a single location, the broad-sense heritability estimates based on plots for each trait across the 11 CN-NAM families were moderately high (Table 1). These estimates of heritability suggested that variations for all of the target traits in the CN-NAM population were primarily influenced through QTLs. The US-NAM population, which included 2948 lines, was phenotyped for only 1 year under the different environments. The US-NAM population represented a validation population and was used to verify the results obtained in the CN-NAM population.

The linkage analysis within a single bi-parental population was successfully used to locate QTLs that affect drought tolerance in maize. In the present study, both NAM populations with much larger mapping population sizes and high-density genetic maps were used to perform the first detection of QTLs that control drought tolerance. A total of 169 joint QTLs were detected under the water-stressed environment in the both NAM populations. These drought-related QTLs were compared with previously reported meta-QTLs (mQTLs) (Additional file 11), which provided a good summary of the published QTLs associated with the drought tolerance-related traits involved in the present study. Among 169 drought-related QTLs, 106 QTLs overlapped with at least one mQTL and six QTLs overlapped with three mQTLs obtained in three different studies. One genomic region on chromosome 1 (43.75–46.55 Mb) harbored one QTL for WSHKW in the CN-NAM population and one QTL for WSKNPR in the US-NAM population, and it also contained three mQTLs that were also reported in the studies of Li et al. [14], Semagn et al. [15] and Almeida et al. [12]. Particularly, Li et al. [14] identified the candidate gene *pdc3* as associated with drought tolerance in this region through a bioinformatics analysis. The region between 204.65 and 206.18 Mb on chromosome 3 included three QTLs for WSHKW, WSEL and WSEW in the CN-NAM population. In this physical region, Almeida et al. [12] detected an mQTL that affects the number of ears per plant under WS conditions, suggesting that this region is an important drought tolerance locus that controls grain yield. Another important genomic region located in the 85.32–95.89 Mb interval on chromosome ten overlapped with three QTLs for WSHKW and WSASI in the CN-NAM population and WSASI in the US-NAM population. Almeida et al. [11] and Li et al. [14] reported two mQTLs in this region. In addition, the gene *ZmSNAC1*, which is responsive to drought stress in maize [27], was located in this region. The genomic regions described above provide important target regions for identifying candidate genes associated with drought stress and marker-assisted introgression for drought tolerance in maize.

The fifty-two candidate genes identified in the present study were identified as encoding transcription factors, signal transduction factors, dehydrins and osmotins, etc. (Table 2). These genes have also been frequently associated with drought tolerance in plants. For example, the GRMZM2G081214 gene was associated with the most highly robust SNP (BPP = 0.95) located in the QTL region. This gene is predicted role in ABA activity and encodes a phosphate-responsive 1 family protein (http://www.maizegdb.org/). The gene is more differentially up-regulated in the ovaries of the inbred line B73 under WS compared with WW.

Two robust associated SNPs were located near the GRMZM2G082855 gene, which is associated with plant height and kernel number per row. This gene encodes the precursor to receptor-like protein kinase 5, a plant protein kinase responsive to abiotic stress. Receptor-like kinase (RLK) is widespread in plants, particularly in *Arabidopsis* and rice, and includes 600 and 1132 RLKs, respectively [28].

Two genes, GRMZM2G143640 and GRMZM5G805609, were associated with the ASI and harbored one strong associated SNP in the ASI QTL regions on chromosome 8, respectively. GRMZM2G143640 encodes a DIVARICATA-like putative MYB DNA-binding domain superfamily transcript factor that plays an important role in plant growth and development and responds to abiotic stress, including drought tolerance [29]. This candidate gene was more differentially up-regulated under WS than WW in the inbred line B73. GRMZM5G805609 encodes glucan endo-1,3-beta-glucosidase 7 of the glycosyl hydrolase protein superfamily, and it was also significantly up-regulated in the inbred line B73 under drought stress.

Most of the remaining genes listed in Table 2 were associated with grain yield component traits, which are highly associated with drought tolerance. A full understanding of the genetic control of these traits would be helpful for maize breeding for high yields under watered-well or water-stressed environments.

**Fig. 3:**
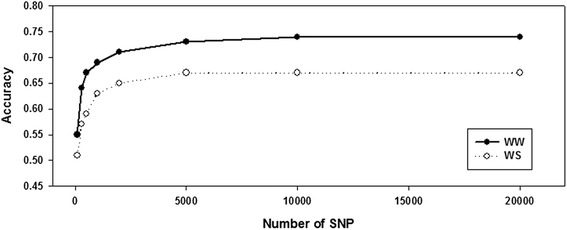
Accuracy of the whole-genome predictions of PH under the WW and WS conditions within the CN-NAM population depending on the number of SNPs. Accuracies averaged over 20 cross-validation runs are shown for 100, 300, 500, 1000, 2000, 5000, 10000 and 20000 evenly spaced SNPs

With the advancements of next-generation sequencing and statistical models, maize breeders have successfully used genomic prediction methods to estimate the breeding value of unphenotyped lines within breeding populations [30], single bi-parental or multiple bi-parental populations [31, 32] and association populations [33]. Genomic prediction models have been demonstrated as advantageous for complex traits controlled through many small-effects loci, such as grain yield [34, 35]. In the present study, good prediction accuracies were obtained for the target traits under WS, although certain traits showed low heritability. We observed that trait heritability affects the prediction accuracy of the CN-NAM population. The target traits with high heritability generally achieved high prediction accuracy under the WS or WW conditions. Marker density has previously been demonstrated as an important factor for prediction accuracy [36, 37]. We selected 100, 300, 500, 1000, 2000, 5000, 10000 or 20000 SNPs that evenly covered the entire genome to investigate the influence of marker density on the prediction accuracy of the CN-NAM population (Fig. 3). The results indicated that approximately 5000 SNP markers were sufficient to achieve accurate predictions for the drought-related traits under the WW or WS environments. Zhang et al. [38] used low-density SNPs and high-density markers (GBS) to predict the grain yield, flowering time and plant height for 19 bi-parental populations under WW and WS treatments, and their results indicate that moderate marker density was largely sufficient for complex and simple traits. Genomic predictions for drought-related traits are efficient in multiple bi-parental populations, such as the CN-NAM population. However, additional studies are needed to assess the prediction accuracy for drought tolerance within association populations with unrelated lines.

## Conclusions

In this study, a total of 220 QTLs under well-watered conditions and 169 QTLs under water-stressed conditions were detected by joint linkage mapping in the CN-NAM and US-NAM populations. The genome-wide association analysis identified 365 SNPs associated with drought-related traits, and these SNPs were located in 354 candidate genes. Of these candidate genes, 52 candidate genes showed significant differential expression in the inbred line B73 under the well-watered and water-stressed conditions. In addition, the moderate-density SNPs obtained through genotyping-by-sequencing were able to make accurate predictions in the nested association mapping population for drought-related traits with moderate-to-high heritability under the water-stressed conditions. This paper presents these drought tolerance QTLs and candidate genes for the maize scientific community to provide detailed direction for future studies.

## Methods

### Plant materials and field environments

The CN-NAM and US-NAM panels were generated as previously described [20]. A total of 1972 CN-NAM lines were grown and measured in the spring of 2009 and 2010 in Urumqi of Xinjiang Province, where the institute of crop science belonging to the Chinese Academy of Agricultural Sciences has set up experimental field bases. The institute of crop science was approved for field experiments, and the field studies did not involve endangered or protected species. For each year, all of the plant materials were subjected to well-watered (WW) and water-stressed (WS) treatments. Trials of each treatment were planted in single-row plots of 11 plants, and there were two replications. Two irrigation regimes were applied using the furrow irrigation method starting at the seeding period. In the WW regime, irrigation was provided in 15-day intervals. In the WS regime, irrigation was given until 3 weeks prior to the expected anthesis date in each CN-NAM family. This stress condition was maintained until 4 weeks after 50 % of the RILs flowered. Nineteen of the 25 US-NAM families, including 2948 RILs, were grown in Urumqi, Xinjiang Province in the spring of 2013. Based on the previous flowering time data [20], four families (CML228, CML247, CML52, and IL14H) with late maturity were excluded from the drought tolerance identification. The P39 and Hp301 families belonging to the sweet and popcorn types were also excluded in the present study. Single-row plots of 11 plants with one replication were grown for each RIL under the WW and WS environments. Each plot was 3 m in length, and the rows were spaced 0.6 m apart. The RIL families were randomly arranged as previously described [39]. Within each family, incomplete blocks consisting of 40 random RILs, the B73 line and alternate parents of the family were planted in an alpha lattice. In the WS regime, the drip irrigation method was applied in 10-day intervals until 3 and 5 weeks prior to the expected anthesis date and after 50 % anthesis occurred. For the WW condition, the soil moisture was maintained at field capacity. All plant materials used in this study were conserved in our experiment lab and we declared that all plant materials used in this study comply with the “Convention on the Trade in Endangered Species of Wild Fauna and Flora”.

### Phenotyping

A total of seven traits were measured for all of the lines under both water regimes. The anthesis-silking interval (ASI) was counted as the difference (in days) between male and female flowering times in each plot. The plant height (PH) was calculated as the average height of five random plants measured from the ground to the tassel tip in each plot. Five representative plants in each plot were harvested. The grain yield per plant (GYPP) was evaluated from an average of five plants. The ear length (EL), kernel number per row (KNPR), and ear weight (EW) were measured for five ears and averaged over the plot. The hundred kernel weight (HKW) was estimated from the average weight of 100 randomly selected seeds in three samples.

### Genotypic data

A set of 0.95 million SNPs from the CN-NAM and US-NAM populations was generated using Genotyping-By-Sequencing technology [40]. The missing SNPs were imputed using the FILLIN method in TASSEL v.5.0 [41]. These marker sets are publicly available at http://www.panzea.org. The SNP sites exhibiting more than 20 % missing taxa, <5 % minor allelic frequency (MAF), and >20 % heterozygosity were excluded from the raw genotype datasets. Thus, a total of 333,577 and 404,543 SNPs were identified for the CN-NAM and US-NAM populations, respectively. These SNPs were subsequently used in the GWAS and genomic predictions. For the joint linkage mapping, two composite genetic maps that included 4932 and 5296 markers were constructed for the CN-NAM and US-NAM populations, respectively. Detailed information on the maps and genotypes scores has previously been described [20].

### Phenotypic data analysis

For the CN-NAM and US-NAM populations, the best linear unbiased prediction (BLUP) for all of the traits of each line across environments (CN-NAM) and within a single environment (US-NAM) was calculated from a random effects model using PROC MIXED in SAS 9.2. In models across the environments of the CN-NAM population, the environment, family, family*environment and entry (family) were considered random effects. The mixed model was fitted within a single environment of the US-NAM population, and the family, RIL within family, blocks, rows and columns were included in the field design. Correlation coefficients were obtained based on the BLUP using Pearson’s statistic applied using the *cor* function of R software. The broad-sense heritability (h^2^) for each trait across environments in the CN-NAM population was calculated on a plot basis using the ANOVA tool in QTL IciMapping Version 3.3 [42].

### Joint linkage mapping in CN-NAM and US-NAM

The joint linkage analysis for CN-NAM and US-NAM was conducted in SAS 9.2. The detailed information for joint linkage mapping has previously been described [21]. Briefly, PROC GLMSelect was implemented to select the significant marker effects in a family-nested QTL model. For all of the traits, the *P*-values for the entry and exit of the model were determined using permutation testing. The phenotypic variation explained by all of the QTLs was counted according to Li [43]. The joint linkage QTL support intervals were counted according to Tian [22].

### GWAS in CN-NAM and US-NAM

The GWAS was performed in a single NAM population using the Fixed and random model Circulating Probability Unification (FarmCPU) method in R software [44]. To identify the SNPs with the most robust associations with phenotypes, a subsampling procedure was used in the GWA analysis [45]. For each subsampling, 80 % of the RILs of each NAM population were randomly sampled without replacement. This procedure was repeated 100 times between each trait and polymorphism. In each subsampling, we implemented a Bonferroni-corrected threshold probability of 0.05/N to verify the significance levels, where N is the number of individual trait-SNP combinations tested. The significance levels were used to control the false positives in the GWAS. The bootstrap posterior probability (BPP), which is defined as the proportion of times that SNPs were included in the 100 subsamples, was calculated for each significantly associated SNP. Only SNPs with BPP ≥ 0.05 were listed in the results. According to Valdar et al. [45], a BPP ≥ 0.25 was considered the most robust SNP association.

### Validation of the candidate genes using RNA-seq data

Based on the maize B73 reference genome assembly V2, genes co-localizing with or adjacent to the associated SNPs were determined to be candidate genes for drought tolerance. Functional annotations of the candidate genes were conducted using blastp, conserved domain search tools, the Maize Genome Database and a literature-specific inspection for each gene. To validate the candidate genes for drought tolerance revealed in the GWAS, the available RNA-seq data of the inbred line B73 was used to evaluate the expression of candidate genes under the WW and WS environments [46]. The RNA-seq data from pollinated ovaries of drought sensitive inbred B73 under WW and WS environments were downloaded from the NCBI web site (http://www.ncbi.nlm.nih.gov/sra/). Briefly, after obtaining the RNA-seq data, the quality control of the raw reads were dealt with the FASTX toolkit (http://hannonlab.cshl.edu/fastx_toolkit/). High-quality RNA-seq reads were mapped to the maize B73 reference genome (B73 AGPv2; http://www.maizesequence.org) using the programs TopHat v2.0.4 [47]. Differential expression analysis was conducted using the HTSeq-DEseq workflow [48]. A false discovery rate (FDR) <0.05 after Benjamini-Hochberg correction for multiple tests was applied.

### Cross-validated genomic prediction

We conducted genomic predictions using a mixed-model solver in the rrBLUP package [49] distributed by R software. The prediction accuracy of all traits was evaluated through cross-validation. To perform the cross-validation, a five-fold cross-validation scheme was applied and repeated 20 times. All of the lines in the NAM panel were randomly divided into five disjointed subsets. One of five subsets was selected as the validation population, and the remaining four subsets were used as the training population to estimate the SNP effects for predicting the lines’ values in the validation. The prediction accuracy was calculated according to Pearson’s correlation between the predicted and observed values averaged over all of the cross-validations and replicates.

## Additional files

Additional file 1: Table S1. Statistical analysis of the seven drought-related traits’ BLUP values under the WW and WS conditions within the CN-NAM population. (DOCX 19 kb)
Additional file 2: Table S2. Statistical analysis of the seven drought-related traits value under WW and WS within the US-NAM population. (DOCX 19 kb)
Additional file 3: Table S3. Correlation of the seven drought-related traits under the WW (above diagonal) and WS (under diagonal) conditions within the CN-NAM population. (DOCX 19 kb)
Additional file 4: Table S4. Correlation of the seven drought-related traits under the WW (above diagonal) and WS (under diagonal) conditions within the US-NAM population. (DOCX 19 kb)
Additional file 5: Joint linkage analysis summary for the seven drought-related traits under the WW and WS conditions in the CN-NAM population. (XLSX 65 kb)
Additional file 6: Joint linkage analysis summary for the seven drought-related traits under the WW and WS conditions in the US-NAM population. (XLSX 79 kb)
Additional file 7: Table S5. Joint QTL validation between the CN-NAM and US-NAM populations under the WW and WS conditions. (DOCX 18 kb)
Additional file 8: Significantly associated SNPs at the threshold of BPP = 0.05 for all of the traits under the WW and WS conditions in the CN-NAM population. (XLSX 126 kb)
Additional file 9: Significantly associated SNPs at the threshold of BPP = 0.05 for all of the traits under the WW and WS conditions in the US-NAM population. (XLSX 83 kb)
Additional file 10: All of the robust SNPs associated with the seven drought-related traits and the closest candidate genes in the two NAM populations. (XLSX 40 kb)
Additional file 11: Comparison between the QTL results identified for the seven drought-related traits under WS in both NAM populations and the reported mQTLs associated with drought tolerance. (XLSX 19 kb)

## Abbreviations

ASI: Anthesis-silking interval
BLUP: Best linear unbiased prediction
BPP: Bootstrap posterior probability
CN-NAM: Chinese nested association mapping
EL: Ear length
EW: Ear weight
GWAS: Genome-wide association studies
GYPP: Grain yield per plant
HKW: Hundred kernel weight
KNPR: Kernel number per row
PH: Plant height
US-NAM: United State nested association mapping
WS: Water-stressed
WW: Well-watered
