# Supplementary material for: Numerous genetic loci identified for drought tolerance in the maize nested association mapping populations
Source: BMC Genomics. 2016 Nov 8;17:894. doi: 10.1186/s12864-016-3170-8 (PMC5101730; doi:10.1186/s12864-016-3170-8)
Supplement: Additional file 1: Table S1. — Statistical analysis of the seven drought-related traits’ BLUP values under the WW and WS conditions within the CN-NAM population. (DOCX 19 kb) [file 12864_2016_3170_MOESM1_ESM.docx]

Table S1. Statistical analysis of seven traits BLUP value under WW and WS within the CN-NAM population

| Trait | Treatment | Average±SD | Range | WS/WW | H^2^ (%) |
| --- | --- | --- | --- | --- | --- |
| ASI | WW | 2.5±1.32 | -1.4-8.1 | 1.48** | 61.7 |
|  | WS | 3.7±1.52 | -4.4-9.9 |  | 57.3 |
| PH | WW | 171.7±18.11 | 112.8-237.8 | 0.91** | 87.2 |
|  | WS | 155.6±16.62 | 98.3-224.4 |  | 75.9 |
| GYPP | WW | 86.0±20.59 | 34.1-198.2 | 0.72** | 68.9 |
|  | WS | 62.0±11.37 | 32.5-119.5 |  | 49.3 |
| EW | WW | 83.2±22.37 | 38.6-225.2 | 0.81** | 79.5 |
|  | WS | 67.2±13.56 | 38.2-149.5 |  | 56.5 |
| EL | WW | 13.1±1.69 | 8.5-19.6 | 0.92** | 81.5 |
|  | WS | 12.1±1.29 | 8.1-18.2 |  | 67.2 |
| KNPR | WW | 22.6±3.31 | 13.1-36.2 | 0.85** | 77.7 |
|  | WS | 19.3±2.53 | 12.6-29.9 |  | 61.3 |
| HKW | WW | 27.7±4.57 | 14.7-46.7 | 0.94** | 89.4 |
|  | WS | 26.3±3.85 | 14.6-41.2 |  | 83.5 |
