# Supplementary material for: Numerous genetic loci identified for drought tolerance in the maize nested association mapping populations
Source: BMC Genomics. 2016 Nov 8;17:894. doi: 10.1186/s12864-016-3170-8 (PMC5101730; doi:10.1186/s12864-016-3170-8)
Supplement: Additional file 2: Table S2. — Statistical analysis of the seven drought-related traits value under WW and WS within the US-NAM population. (DOCX 19 kb) [file 12864_2016_3170_MOESM2_ESM.docx]

Table S2. Statistical analysis of seven traits BLUP value under WW and WS within the US-NAM population

| Trait | Treatment | Average±SD | Range | WS/WW |
| --- | --- | --- | --- | --- |
| ASI | WW | 3.6±2.19 | -4-12 | 1.64** |
|  | WS | 5.9±3.55 | -8-20 |  |
| PH | WW | 213.4±26.04 | 101.6-311.6 | 0.85** |
|  | WS | 180.7±22.79 | 85.3-270.6 |  |
| GYPP | WW | 73.6±31.07 | 1.3-197.3 | 0.34** |
|  | WS | 25.3±20.78 | 1.1-129.7 |  |
| EW | WW | 104.4±33.06 | 11.1-238.9 | 0.52** |
|  | WS | 54.6±26.92 | 7.0-149.1 |  |
| EL | WW | 14.6±2.17 | 7.2-21.6 | 0.83** |
|  | WS | 12.1±2.09 | 6.9-19.8 |  |
| KNPR | WW | 21.2±5.26 | 4.0-39.2 | 0.65** |
|  | WS | 13.9±4.68 | 2.0-36.5 |  |
| HKW | WW | 25.1±4.48 | 12.4-41.2 | 0.88** |
|  | WS | 22.3±4.14 | 8.0-39.3 |  |
