# Supplementary material for: Numerous genetic loci identified for drought tolerance in the maize nested association mapping populations
Source: BMC Genomics. 2016 Nov 8;17:894. doi: 10.1186/s12864-016-3170-8 (PMC5101730; doi:10.1186/s12864-016-3170-8)
Supplement: Additional file 3: Table S3. — Correlation of the seven drought-related traits under the WW (above diagonal) and WS (under diagonal) conditions within the CN-NAM population. (DOCX 19 kb) [file 12864_2016_3170_MOESM3_ESM.docx]

Table S3. Correlation for seven traits under WW (above diagonal) and WS (under diagonal) within CN-NAM

| trait | ASI | EL | GYPP | KNPR | HKW | PH | EW |
| --- | --- | --- | --- | --- | --- | --- | --- |
| ASI | 1 | -0.01 | -0.24^**^ | -0.08^**^ | -0.25^**^ | -0.15^**^ | -0.16^**^ |
| EL | 0.004 | 1 | 0.49^**^ | 0.61^**^ | 0.26^**^ | 0.24^**^ | 0.63^**^ |
| GYPP | -0.17^**^ | 0.49^**^ | 1 | 0.64^**^ | 0.36^**^ | 0.36^**^ | 0.72^**^ |
| KNPR | -0.08^**^ | 0.60^**^ | 0.72^**^ | 1 | -0.11^**^ | 0.19^**^ | 0.58^**^ |
| HKW | -0.19^**^ | 0.19^**^ | 0.23^**^ | -0.13^**^ | 1 | 0.16^**^ | 0.49^**^ |
| PH | -0.12^**^ | 0.25^**^ | 0.31^**^ | 0.20^**^ | 0.06^**^ | 1 | 0.30^**^ |
| EW | -0.11^**^ | 0.62^**^ | 0.70^**^ | 0.65^**^ | 0.36^**^ | 0.26^**^ | 1 |

*, ** Significant at *P*≤0.05, 0.01, respectively
