# Supplementary material for: Numerous genetic loci identified for drought tolerance in the maize nested association mapping populations
Source: BMC Genomics. 2016 Nov 8;17:894. doi: 10.1186/s12864-016-3170-8 (PMC5101730; doi:10.1186/s12864-016-3170-8)
Supplement: Additional file 4: Table S4. — Correlation of the seven drought-related traits under the WW (above diagonal) and WS (under diagonal) conditions within the US-NAM population. (DOCX 19 kb) [file 12864_2016_3170_MOESM4_ESM.docx]

Table S3. Correlation for seven traits under WW (above diagonal) and WS (under diagonal) within US-NAM

| trait | ASI | EL | GYPP | KNPR | HKW | PH | EW |
| --- | --- | --- | --- | --- | --- | --- | --- |
| ASI | 1 | -0.04 | -0.34^**^ | -0.18^**^ | -0.14^**^ | 0.08^**^ | -0.23^**^ |
| EL | -0.02 | 1 | 0.51^**^ | 0.72^**^ | 0.14^**^ | 0.26^**^ | 0.66^**^ |
| GYPP | -0.42^**^ | 0.50^**^ | 1 | 0.64^**^ | 0.23^**^ | 0.19^**^ | 0.80^**^ |
| KNPR | -0.26^**^ | 0.69^**^ | 0.74^**^ | 1 | 0.20^**^ | 0.16^**^ | 0.74^**^ |
| HKW | -0.11^**^ | 0.19^**^ | 0.25^**^ | -0.16^**^ | 1 | 0.17^**^ | 0.31^**^ |
| PH | -0.07^**^ | 0.22^**^ | 0.20^**^ | 0.15^**^ | 0.21^**^ | 1 | 0.23^**^ |
| EW | -0.31^**^ | 0.62^**^ | 0.85^**^ | 0.84^**^ | 0.31^**^ | 0.21^**^ | 1 |

*, ** Significant at *P*≤0.05, 0.01, respectively
