# Supplementary material for: Numerous genetic loci identified for drought tolerance in the maize nested association mapping populations
Source: BMC Genomics. 2016 Nov 8;17:894. doi: 10.1186/s12864-016-3170-8 (PMC5101730; doi:10.1186/s12864-016-3170-8)
Supplement: Additional file 7: Table S5. — Joint QTL validation between the CN-NAM and US-NAM populations under the WW and WS conditions. (DOCX 18 kb) [file 12864_2016_3170_MOESM7_ESM.docx]

Table S4. QTL validation between CN-NAM and US-NAM under WW and WS

| Trait | WW | | |  | WS | | |
| --- | --- | --- | --- | --- | --- | --- | --- |
|  | QTL number of CN-NAM | QTL number of US-NAM | Shared QTL between two NAM |  | QTL number of CN-NAM | QTL number of US-NAM | Shared QTL between two NAM |
| ASI | 15 | 19 | 4 |  | 11 | 7 | 3 |
| PH | 21 | 25 | 5 |  | 20 | 20 | 6 |
| GYPP | 8 | 10 | 1 |  | 8 | 9 | 1 |
| EL | 15 | 17 | 6 |  | 11 | 12 | 3 |
| HKW | 23 | 20 | 7 |  | 18 | 13 | 4 |
| KNPR | 13 | 11 | 5 |  | 10 | 8 | 0 |
| EW | 13 | 10 | 4 |  | 13 | 9 | 1 |
| Total | 108 | 112 | 32 |  | 91 | 78 | 18 |
